# The Treatment of Parenchymatous Goitre

**Published:** 1929

**Authors:** W. A. Jackman

**Affiliations:** Assistant Surgeon to the Bristol Royal Infirmary; Assistant Surgeon to the Royal Hospital for Women and Children


### THE TREATMENT OF PARENCHYMATOUS GOITRE.

BY

W. A. Jackman, M.B., Ch.B., F.R.C.S.E.,

*Assistant Surgeon to the Bristol Royal Infirmary; Assistant Surgeon to the Royal Hospital for Women and Children.*

A rational introduction to the subject of "The Treatment of Parenchymatous Goitre" demands, at least, a brief consideration of the pathology of this condition. Effective treatment must have as a basic principle the correction and removal of the factors responsible for producing the diseased state, and in addition it is usually found that the organ or organs affected demand rest, physical, physiological, or both.

The ætiology of parenchymatous goitre has been discussed *ad nauseam*, and it is only proposed here to pick out of the medley of theoretical observations a few of the facts that seem to be established with reasonable certainty.

About the age of puberty the body calls for some readjustment of the endocrine system generally, and during this process it is probable that a considerably increased demand is made upon the thyroid gland for additional secretion. In normal individuals the gland responds readily, and is able to supply sufficient secretion for the needs of the body, without hypertrophy.

In certain persons the thyroxin is deficient in amount or lacking in quality, or for some obscure reason the body is unable to make use of it with customary economy. In its effort to deal with the situation the gland enlarges. Even in apparently normal persons the gland enlarges slightly when it has to work overtime, the most conclusive evidence being the enlargement of the thyroid gland often seen during menstruation, and its return to normal during the intervening stages. This is often more evident during the early years following the onset of the periods, and becomes less apparent as age advances. May we not regard this occurrence as a step on the road to the formation of a parenchymatous goitre, and suppose that it only needs an unknown addition or subtraction to disorganize the general endocrine balance or to make it more difficult for the body to utilize the thyroid secretion ?

This unknown quantity would appear to be produced by some obscure form of infection. It is well known that drinking water has been blamed, and that there is definite evidence in support of this. However, one cannot disregard the possibility of auto-intoxication as an additional or even the sole determining factor.

The common pathological change found in the true parenchymatous goitre associated with puberty is a general hypertrophy and hyperplasia of all the elements of the thyroid gland, including, as might reasonably be expected, an excess of colloid material within the acini. In some cases one or more acini become distended to such an extent that they form obvious cystic swellings on the surface of the gland.

The further changes associated with hemorrhage, excessive vascularity, predominance of fibrous tissue and other complications do not come within the scope of this paper ; their treatment depends upon the special indication of each particular variety.

The evidence gleaned from the microscope and from observation of the patients must lead to the deduction that the tissues, in parenchymatous goitre, are calling for more thyroxin and for the removal of that which prevents its efficient usage. The thyroid hypertrophies in order to cope with the increased demand upon its resources. Removal of any part of the gland, under these circumstances, would appear to be directly contra-indicated, yet in standard text-books and in many papers on this subject operation is advised on somewhat slender grounds.

Treatment by administration of iodides or iodine, X-rays or even thyroid extract is mentioned, but in few instances are any details given as to the method or length of time of therapy on these lines.

Pressure symptoms or signs of toxicity are invariably cited as presenting a definite indication for removal of part of the gland, yet the pathology indicates the need for more thyroid still. The fact that extensive fibrosis of the gland following this disease results in myxœdema should surely stay the hand of the most enthusiastic operator, except in the presence of alarming symptoms. Removal is so comparatively easy and calls for so much less patience and care, that recommendation of this form of treatment is apt to be made before the more tedious remedies have had a fair trial.

The following is an outline of the treatment which has a reasonable claim to be based upon the pathology of parenchymatous goitre.

A correct diagnosis having been made, the patient is examined carefully for any focus of infection. Their name is legion, and it is difficult to enumerate any without causing comment from various specialists, in whose department the possibility of a focus is not mentioned. However, there are two sites worthy of special consideration: the mouth, carious teeth and infected tonsils; the nasopharynx, infected adenoids. These foci should be efficiently treated. The patient or her parents should be interrogated in regard to the water supply, although this, more often than not, is merely of theoretical interest. The drinking water should be boiled or obtained from another source if of doubtful origin.

Treatment by administration of thyroid extract should be commenced at once, whatever additional measures it is thought advisable to take.

The thyroid extract is given in tablet form and is obtained from a firm of repute. The doses mentioned are the equivalent of the fresh gland. The age or sex of the patient does not influence the dosage, which, as will be seen, is entirely dependent on the effect produced.

The initial dose is one grain three times a day, after meals. This is increased to two grains at the end of a week, to three grains three times a day at the end of a fortnight, and the increase is maintained in this way, if necessary, up to the limit of the patient's tolerance. It is found, as might be expected, that considerable caution is essential when large doses are reached. When a patient is taking twenty-one or more grains every day it is often advisable not to increase the dose further for three or four weeks. As soon as the dose reaches twelve or fifteen grains a day the goitre commences to decrease in size, in most cases. An optimum dose is usually reached without the necessity of producing symptoms of poisoning.

The estimation of an optimum dose is a matter dependent upon the doctor's own judgment and common sense, but a steady diminution in the size of the goitre may be taken as a good indication that no further increase in the dose is required. The treatment should be continued until the neck appears normal, and the dose should then be decreased by the same method as that by which it was increased in the initial stages. The patient should be watched carefully during the process of reduction of the dose, and if any undue re-enlargement of the gland should occur the amount should be raised again. The average amount of thyroid extract required to cure a parenchymatous goitre, excluding the periods of increasing to and decreasing from the optimum dose, is twenty-one grains a day for six weeks to two months. The full course of treatment seldom takes less than six months, and quite frequently longer.

Strict attention to detail is necessary to attain any degree of success.

In addition to any notes which are made when the case is first seen, the following particulars should be noted and a record kept of them each time the patient attends :—

1. Measurement of the neck. This may be misleading, for several reasons : (*a*) because the patient is growing ; (*b*) because the neck may not be measured at the same level on each occasion ; (*c*) because the measurement may be taken sometimes during menstruation. Inquiry should always be made in regard to this, as these patients almost always do have enlargement of the thyroid gland at these times, which persists even after the goitre is cured.

The following points are helpful in obtaining accurate measurement. The patient should always be seated. The tape should be drawn once or twice across the back of the neck and kept as horizontal as possible, when it will be found to settle in the same place each time. Take the horizontal measurement and the greatest possible measurement. Record both. Do not fail to be guided also by the general appearance of the neck, in this respect the opinion of the patient herself is of the utmost value.

2. Inquiry regarding headaches. The onset of persistent headache in a patient hitherto free from this disability is a most valuable early sign of over-dosage.

3. Pulse-rate. This should be taken and recorded at each visit. A marked and continued rise may call for reduction of the dose of thyroid extract. Needless to say, recent exertion or nervousness must be taken into account.

4. Fine tremor of the extended hands, and

5. Exaggerated knee-jerks demand a re-survey of the case and probably a temporary decrease of the dose.

The necessity for economizing time was responsible for the choice of the foregoing motley collection of signs. They have, however, proved valuable, and if the patient be instructed to report any fresh symptom immediately, there need be little fear of administering a poisonous dose of thyroxin or of neglecting to provide more orthodox treatment, should this be failing. By adopting these signs as a guide, it is not necessary to spend more than five minutes with the patient at each attendance subsequent to the first examination.

Up to the present time twenty-nine cases have been treated in this way by the author, with one complete failure and one doubtful result. The failure was a case in which the patient had already had part of her goitre removed, and the remaining thyroid, still struggling on to do its best, had hypertrophied again. The patient evidently got tired of the treatment and ceased to attend, but she never showed any sign of improvement. The doubtful result is a man aged twenty-five years, who cannot tolerate large doses of thyroid. He has had a series of small doses over a prolonged period, and his goitre is considerably smaller and softer, but it has become necessary to cease giving the thyroid. He is now taking syrupus ferri iodidi, and maintains his improvement.

By far the majority of the cases treated by this method have been girls between the ages of eleven and eighteen years, and it is for these patients that the treatment is most suitable. Most of the cases may be treated as out-patients, but cases with pressure symptoms or large cysts should be admitted to a hospital or nursing home and under constant observation raised rapidly to the optimum dose. One case in particular deserves special mention.

A. B., a girl aged 12 years, came to the Bristol Children's Hospital with a large goitre, slight pressure symptoms, two obvious cysts, some exophthalmos and tachycardia. On account of her age and the size of the goitre it was decided to treat her as a parenchymatous goitre of puberty. She was admitted to hospital and kept in bed. The dose of thyroid extract was increased from three to thirty grains a day in under a fortnight. As the dose was increased her pulse-rate diminished, the exophthalmos became less marked, the goitre decreased in size and the cysts disappeared. In a month she was fit for discharge to the out-patient department, where her treatment was continued until she was permanently cured. Her sister suffered also from a goitre, but her symptoms were not so severe. They both derived their supply of drinking water from a Gloucestershire well. Her parents, who did not live in the district until well past the age of puberty, had no sign of goitre.

This case is mentioned, not only on account of its peculiar interest, but also to demonstrate the possible efficiency of the treatment, even in cases associated with dysthyroidism.

It is considered that the treatment is not suitable for cases of long standing, in whose thyroid glands permanent pathological changes have taken place. The cases which yield most rapidly are those in whom menstruation has not started at the time the goitre is first seen and the treatment commenced.

A learned physician, to whom the plan was unfolded, said that he did not think that the method would be harmful, but a waste of time and of thyroid extract, as the cases all cured themselves. If this were true, surely we should not see so many patients of more mature years with goitres left over from puberty. Why should patients with goitres obviously becoming worse, with symptoms of increasing pressure, show almost immediate improvement when treated in this way ?

Two stimuli are responsible for the publication of this paper. One is that I am convinced of the need for thyroid in these patients. The other is that the haphazard statement, " thyroid extract may be tried," seen in most text-books, is a totally inadequate description of the treatment, and is responsible for its poor reputation as a remedy for this disease.

I feel I owe an apology to my surgical colleagues for embarking on so medical a subject, and to the physician for encroaching on his perquisites. My excuse is that in the early days of my special interest in this subject these cases were sent and even returned to me by physicians for operation. The cases are alive and well to-day without operation and without goitres. I had a firm belief in this treatment, at first on theoretical grounds, and later from practical experience. I found that I had to carry it out myself or leave it untried. I sincerely trust that this somewhat poor and sketchy account will stimulate others to give the method a trial, as I am convinced of its efficiency.

